# PILRA serves as a diagnostic and prognostic biomarker and modulates the tumor immune microenvironment and immunotherapy response in breast cancer

**DOI:** 10.3389/fimmu.2025.1682519

**Published:** 2026-01-16

**Authors:** Bowen Shi, Yan Yang, Lei Xing, Junxia Chen

**Affiliations:** 1 Department of Breast and Thyroid Surgery, The First Affiliated Hospital of Chongqing Medical University, Chongqing, China; 2 Department of Cell Biology and Genetics, Chongqing Medical University, Chongqing, China

**Keywords:** biomarker, breast cancer, immunotherapy, PILRa, prognosis

## Abstract

**Background:**

Paired immunoglobulin-like type 2 receptor alpha (PILRA) is a membrane-associated receptor involved in immune regulation and signal transduction. However, its expression and functional role in breast cancer remain largely unknown. This study investigated the expression, mutation, and DNA methylation patterns of PILRA in breast cancer, along with its impact on immune infiltration and associated pathways. We also evaluated its potential as a therapeutic target for predicting prognosis and guiding immunotherapy in breast cancer.

**Methods:**

PILRA expression in breast cancer was analyzed using TCGA and GTEx datasets. Protein expression in breast cancer and adjacent normal tissues was evaluated by immunohistochemistry, and expression levels were validated by RT-qPCR in 50 paired tumor and adjacent tissue samples. cBioPortal was used to assess mutation profiles and prognostic relevance. Associations with drug resistance were examined by analyzing relationships to resistance- and sensitivity-related genes. DNA methylation and its prognostic impact were analyzed using MethSurv. The prognostic and diagnostic value of PILRA was evaluated through survival and ROC curve analyses. Single-cell and tissue expression data were obtained from HPA and GTEx, and gene effect score from DepMap. Immune associations were assessed using TISIDB. Gene correlation and protein-protein interaction networks were analyzed via TCGA and STRING, followed by KEGG and GO enrichment.

**Results:**

PILRA expression was upregulated in breast cancer tissues and associated with poor survival and drug resistance. We identified R236M as the dominant mutation site and found that its mutation is linked to improved prognosis. PILRA methylation downregulated its expression and correlated with better prognosis. Survival analysis and ROC curves supported the potential of PILRA as a prognostic biomarker. PILRA was involved in immune infiltration and modulated the abundance of various immune cells and the tumor microenvironment, suggesting a role in immune regulation and tissue maintenance. Correlation and enrichment analyses revealed that PILRA-associated genes were mainly involved in cancer-related processes and pathways, with key hub genes in the PPI network.

**Conclusion:**

We identified PILRA as a diagnostic and prognostic biomarker in breast cancer and analyzed its association with immunotherapy response. The findings provide new insight and potential strategies for breast cancer diagnosis and treatment.

## 1 Introduction

Breast cancer is the most prevalent malignancy among women and the leading cause of cancer-related mortality in females worldwide. In 2020, it surpassed lung cancer as the most commonly diagnosed cancer globally, representing a major global health burden (1). An estimated 2.3 million new cases were reported, accounting for 11.7% of all cancer diagnoses (2). Despite substantial advancements in diagnosis and treatment, the incidence and mortality of breast cancer remain persistently high. The emergence of novel targeted therapies has underscored the importance of molecular biomarkers as essential tools for breast cancer diagnosis, prognosis, therapeutic response prediction, and disease monitoring (3). Thus, discovering reliable prognostic biomarkers is crucial for improving breast cancer management.

Immunotherapy, which is considered the third major breakthrough in cancer treatment, utilizes the host immune system to detect and eradicate malignant cells based on the immune surveillance theory (4). It has shown promising results in malignancies such as melanoma, lung cancer, and leukemia. However, its application in breast cancer remains limited, and research has mainly focused on tumor vaccines and monoclonal antibodies, most of which are still undergoing preclinical or clinical evaluation (5). In the era of precision oncology, identifying novel immune-related biomarkers is essential for predicting patient prognosis and optimizing immunotherapeutic strategies.

Paired immunoglobulin-like type 2 receptor alpha (PILRA) is a transmembrane protein of the immunoglobulin superfamily that is composed of an extracellular Ig-like domain, a hydrophobic transmembrane region, and a short cytoplasmic tail. The extracellular domain mediates ligand binding (e.g., NCAM), and the cytoplasmic domain contains two immunoreceptor tyrosine-based inhibitory motifs (ITIMs) that initiate inhibitory signaling by recruiting SHP-1 and SHP-2 phosphatases following ligand recognition (e.g., CD99, CD8A) (6). PILRA, together with the activating receptor PILRB, forms a receptor pair involved in modulating immune responses and has been implicated in tumor immune regulation. PILRA is associated with the development of immune-related diseases, such as rheumatoid arthritis and Alzheimer’s disease (7, 8). It is highly expressed in immune cells, including macrophages and granulocytes, and participates in immune activation, apoptosis, and signal transduction (9, 10). PILRA modulates T cell and NK cell activity, thereby contributing to immune homeostasis. In the tumor microenvironment, this receptor may exert immunosuppressive effects that facilitate immune evasion and cancer progression. Expression of PILRA in the nervous system further suggests involvement in neurodevelopment. PILRA regulates integrin signaling and inhibits CD99-mediated monocyte migration (11), maintains CD8+ T cell quiescence by interacting with CD8A (12), and suppresses neutrophil expansion via modulation of integrin activation during inflammation (13). However, the prognostic and immunological roles of PILRA in breast cancer remain largely unexplored.

In this study, we performed a comprehensive multi-omics analysis to investigate the expression, prognostic value, and potential immunological role of PILRA in breast cancer. We also investigated its correlation with the tumor immune microenvironment and immunotherapeutic responsiveness to assess its potential as a novel biomarker for breast cancer immunotherapy.

## 2 Materials and methods

### 2.1 Data collection methods and bioinformatic tools

#### 2.1.1 Analysis of the expression pattern of PILRA

The genomic and epigenomic data of PILRA and related clinical information of 33 common cancer types were downloaded from the Cancer Genome Atlas (TCGA, https://portal.gdc.cancer.gov/). PILRA gene expression data from normal tissues were obtained from the publicly available genotype-tissue expression database (GTEx, http://commonfund.nih.gov/GTEx). The comparisons between cancerous and adjacent normal tissues were performed using both TCGA and GTEx datasets. TNMplot (https://tnmplot.com/analysis/) was used to analyze and present the differences in mRNA levels of PILRA between breast cancer tissues and adjacent normal tissues using RNA-Seq data (14).

#### 2.1.2 PILRA mutation status in breast cancer

Pan-cancer analysis of the PILRA genomic alteration landscape, including mutations, amplifications, and deep deletions, was performed using the Cancer Types Summary module of the cBioPortal online platform (https://www.cbioportal.org (15–17). Survival differences between PILRA-altered and -unaltered groups in breast cancer were analyzed.

#### 2.1.3 Correlation analysis of PILRA and drug resistance in breast cancer

Drug response data (AUC values) were obtained from the Cancer Therapeutics Response Portal (CTRP, https://portals.broadinstitute.org/ctrp/). Expression data of PILRA and drug resistance or sensitivity–related genes in breast cancer were obtained from TCGA. Pearson correlation analysis was performed in R to evaluate the association between PILRA expression and both drug response and these genes.

#### 2.1.4 PILRA methylation analysis in breast cancer

The methylation status of PILRA in breast cancer was analyzed using TCGA data through the MethSurv tool (https://biit.cs.ut.ee/methsurv/), and Kaplan–Meier survival analysis was performed to assess the relationship between PILRA methylation sites and patient survival. cBioPortal was used to analyze the correlation between PILRA methylation and its expression in breast cancer patients.

#### 2.1.5 Relationship between PILRA and prognosis in breast cancer patients

The relationship between PILRA expression and overall survival (OS), distant metastasis-free survival (DMFS), and relapse-free survival (RFS) in breast cancer patients was analyzed using the KMPlot website (https://kmplot.com). UALCAN (https://ualcan.path.uab.edu) was used to examine the correlation between PILRA expression and survival across different patient subgroups. Finally, the diagnostic potential of PILRA in pan-cancer was assessed by calculating the area under the ROC curve (AUC) based on TCGA datasets and tissue samples from breast cancer patients.

#### 2.1.6 Cell expression analysis of PILRA in breast tissue

The HPA database (https://www.proteinatlas.org) was used to perform a comprehensive analysis of PILRA in breast tissue, including single-cell analysis, tissue cell analysis, and marker analysis for both cell types. PILRA expression was analyzed in breast cancer cell lines and the GTEx database was used for tissue cell analysis. The gene effect score of PILRA was analyzed using the DepMap database.

#### 2.1.7 The relationship between PILRA expression and immunotherapy

The TISIDB tool (http://cis.hku.hk/TISIDB) was used to analyze the impact of PILRA expression on the abundance of major immune cell subtypes and its association with immune subtypes across various human cancers. Additionally, we specifically examined the relationship between PILRA expression and immune subtypes in breast cancer. The correlation between PILRA expression and the abundance of eight immune cell types, including CD8+ T cells, macrophages, and natural killer (NK) cells.

#### 2.1.8 Functional enrichment analysis of PILRA in breast cancer

The STRING platform (https://string-db.org) was used to plot the protein–protein interaction (PPI) network diagrams for PILRA (18). Gene Set Enrichment Analysis (GSEA) on PILRA was conducted to enrich and visualize related Gene Ontology (GO) terms and Kyoto Encyclopedia of Genes and Genomes (KEGG) pathways.

### 2.2 Sample collection

A total of 50 breast cancer tissues and adjacent tumor tissues were subjected to pathological examination between June 2025 and July 2025 at the First Affiliated Hospital of Chongqing Medical University. The clinicopathological characteristics of patients are summarized in **Table 1**. No preoperative radiotherapy or surgical resection was performed. All specimens were promptly stored in liquid nitrogen after surgery for preservation. The study was approved by the Ethics Committee of Chongqing Medical University. Informed consent was obtained from each patient prior to surgery.

**Table 1:** Correlation between PILRA expression and clinicopathological features in 50 BC patients.

Characteristic		All cases	PILRA		Chi-square	P value
			high	low		
All cases		50	39	11		
Age	<50	15	5	10	20.43	<0.001***
	≥50	35	34	1		
T stage	T1	16	8	8	7.57	0.006**
	T2-3	34	31	3		
N stage	N0	27	17	10	3.97	0.046*
	N1-3	23	20	3		
TNM stage	I	9	3	6	14.21	0.001**
	II/III	41	36	5		

*P < 0.05, **P < 0.01, ***P < 0.001.

#### 2.2.1 RNA extraction from tissue

Fresh tissue samples were rapidly frozen in liquid nitrogen and thoroughly homogenized to prevent RNA degradation. The tissue was then lysed using TRIzol reagent, followed by phase separation with chloroform to extract RNA. The RNA was precipitated with isopropanol, collected by high-speed centrifugation, and washed with 75% ethanol to remove impurities. The RNA pellet was air-dried and dissolved in RNase-free water. Finally, RNA concentration and purity were measured using a spectrophotometer.

#### 2.2.2 Real-time polymerase chain reaction

After total RNA extraction and assessment of concentration and purity, RNA was reverse transcribed into complementary DNA (cDNA) using a reverse transcription reagent. Real-time quantitative PCR (RT-qPCR) was then performed using specific primers and the fluorescent dye SYBR Green. The primers for PILRA were as follows: Forward (F) 5’-AGTCTGTGTATTTCTGCCGAGTTG-3’ and Reverse (R) 5’-AGCCTGGGTGATGGAGAGTTTG-3’. The primers for the housekeeping gene GAPDH were as follows: Forward (F) 5’-GAAGGTGAAGGTCGGAGTC-3’ and Reverse (R) 5’-GAAGATGGTGATGGGATTTC-3’. The PCR reaction mix included cDNA template, specific primers, SYBR Green dye, reaction buffer, and DNA polymerase. The amplification program consisted of an initial denaturation step, followed by amplification cycles, and melt curve analysis. Fluorescence signals were detected in real time during each amplification cycle. Finally, the relative expression levels of the target gene were calculated using the ΔΔCt method, and data were normalized for analysis.

### 2.3 Immunohistochemistry

Tissue samples were fixed in formalin or other fixatives, dehydrated, embedded in paraffin, and sectioned to obtain thin slices. Antigen retrieval was performed by heating or enzymatic treatment to restore antigen epitopes. After application of blocking agents to minimize non-specific binding and reduce background noise, sections were incubated with primary antibodies targeting bind target antigens, followed by incubation in labeled secondary antibodies and enzymatic or fluorescent dye development. Staining results were visualized under a microscope and analyzed qualitatively using image analysis software.

### 2.4 Western blot

Total protein was extracted from different breast cancer cell lines using RIPA lysis buffer supplemented with protease inhibitors. Protein concentration was determined using the BCA assay, and equal amounts of protein were separated by SDS–PAGE and transferred onto PVDF membranes. The membranes were blocked with 5% non-fat milk for 1 h at room temperature and then incubated with a primary antibody against PILRA at 4 °C overnight. After incubation with the appropriate HRP-conjugated secondary antibodies, protein bands were visualized using an enhanced chemiluminescence (ECL) detection system. β-actin was used as the loading control.

### 2.5 Statistical analysis

Continuous variables were summarized as means ± SD or medians with IQRs based on the Shapiro–Wilk normality test. Categorical variables were reported as percentages. Differences in gene expression between groups were assessed using the Wilcoxon rank-sum test or Kruskal–Wallis test. Survival differences were compared by the log-rank test (19, 20). Pearson or Spearman correlation coefficients were used to evaluate associations between continuous variables, including correlations between PILRA and drug resistance–related genes in TCGA. Survival outcomes (OS, DMFS, and RFS) were estimated using Kaplan–Meier curves, with hazard ratios derived from Cox proportional hazards models. The prognostic value of PILRA methylation sites was assessed similarly. ROC curve analysis was used to evaluate the diagnostic performance of PILRA. Functional enrichment was performed using GSEA. All statistical tests were two-sided, and p < 0.05 was considered statistically significant.

## 3 Result

### 3.1 PILRA expression is significantly upregulated in breast cancer compared to normal tissues

**Figure 1:**
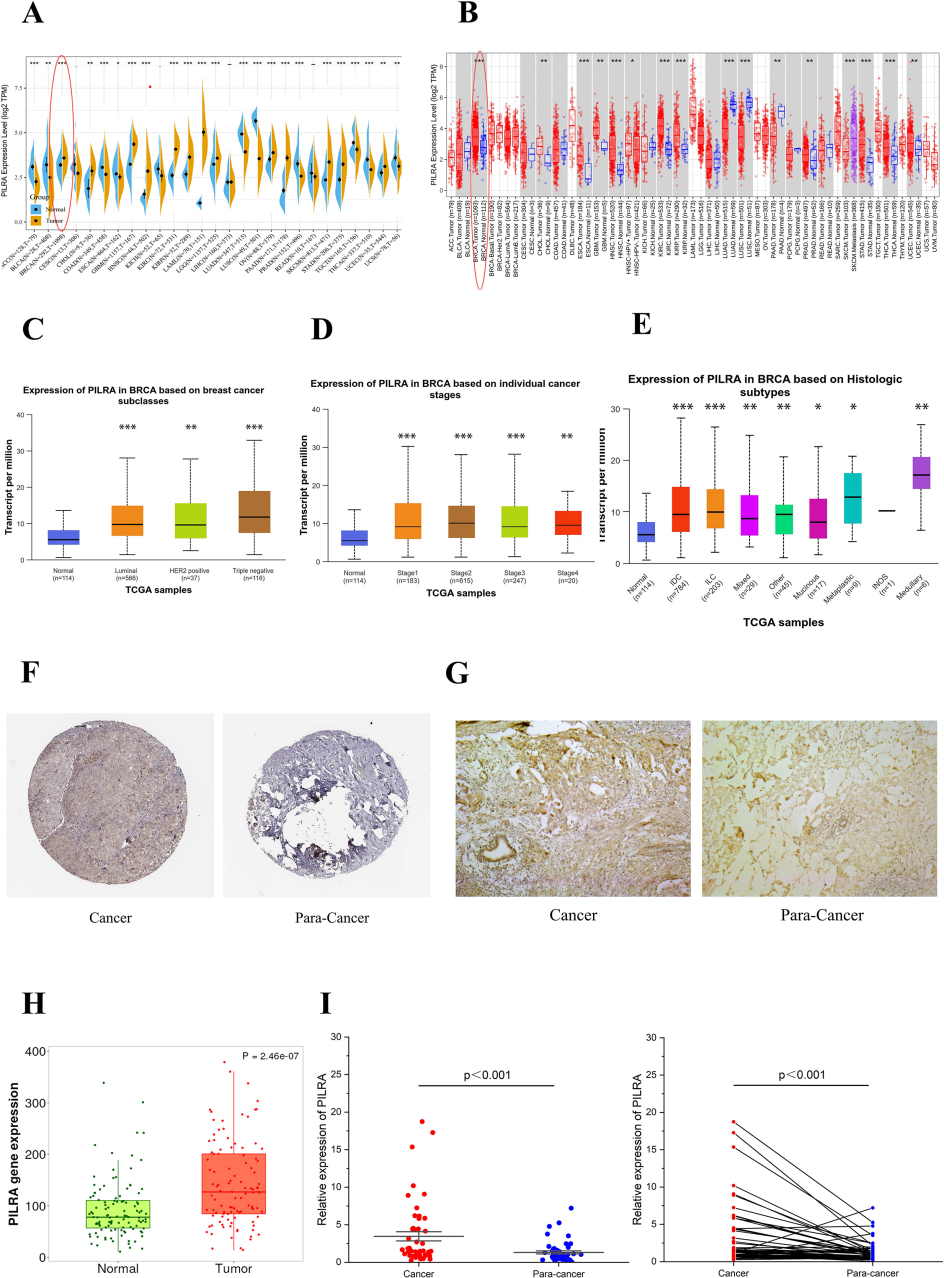
Differential expression analysis of PILRA between breast cancer and normal tissues. **(A)** Bean plots were generated to compare PILRA expression in tumor tissues from TCGA datasets with that in normal tissues from GTEx datasets. **(B)** The differences in PILRA mRNA expression between tumor and normal tissues across 33 cancer types were analyzed using the TIMER2.0 online tool based on TCGA datasets. Gene expression levels were visualized through box plots, with red representing cancerous tissues and blue representing normal tissues. **(C-E)** PILRA expression levels varied across different breast cancer subclasses, individual cancer stages, and histologic subtypes. Asterisks indicate the significance of pairwise comparisons between the normal group and each tumor subtype (P < 0.05; *P < 0.01; **P < 0.001) **(F)** Immunohistochemical staining of PILRA in breast cancer and normal tissues from the THPA database. **(G)** PILRA protein levels in tumor and corresponding normal tissues of breast cancer patients were evaluated by IHC. **(H)** Upregulation of PILRA in breast cancer compared with adjacent normal tissues was analyzed using RNA-seq data from TNMplot. **(I)** RT-qPCR was used to detect the expression of PILRA in breast cancer and para-cancer tissues.

The pan-cancer analysis showed that PILRA expression levels varied significantly between tumor and normal tissues across multiple cancer types (16 out of 23). PILRA expression markedly increased in tumor tissues compared with normal tissues in most cancer types (**Figures 1A, B**). Consistently, PILRA was significantly upregulated in breast cancer. Breast cancer patients were divided into groups to investigate the expression levels of PILRA in different types of patients (**Figures 1C–E**). The stratification was based on several clinical parameters, including cancer subtype, histological subtype, and tumor stage. PILRA expression levels varied across different breast cancer subclasses, histologic subtypes, and individual cancer stages. The protein expression levels of PILRA in breast cancer tissues and normal tissues were analyzed using data from the Human Protein Atlas (THPA) platform (**Figure 1F**). Immunohistochemical analysis (**Figure 1G**) showed that PILRA expression was significantly higher in breast cancer tissues than in normal breast tissues, and analysis of the mRNA expression (**Figure 1H**) revealed elevated PILRA expression in breast cancer tissues. RT-qPCR results from patient samples (**Figure 1I**) confirmed that PILRA expression was significantly higher in breast cancer tissues than in normal breast tissues.

### 3.2 Genetic alterations of PILRA correlate with prognosis in breast cancer

**Figure 2:**
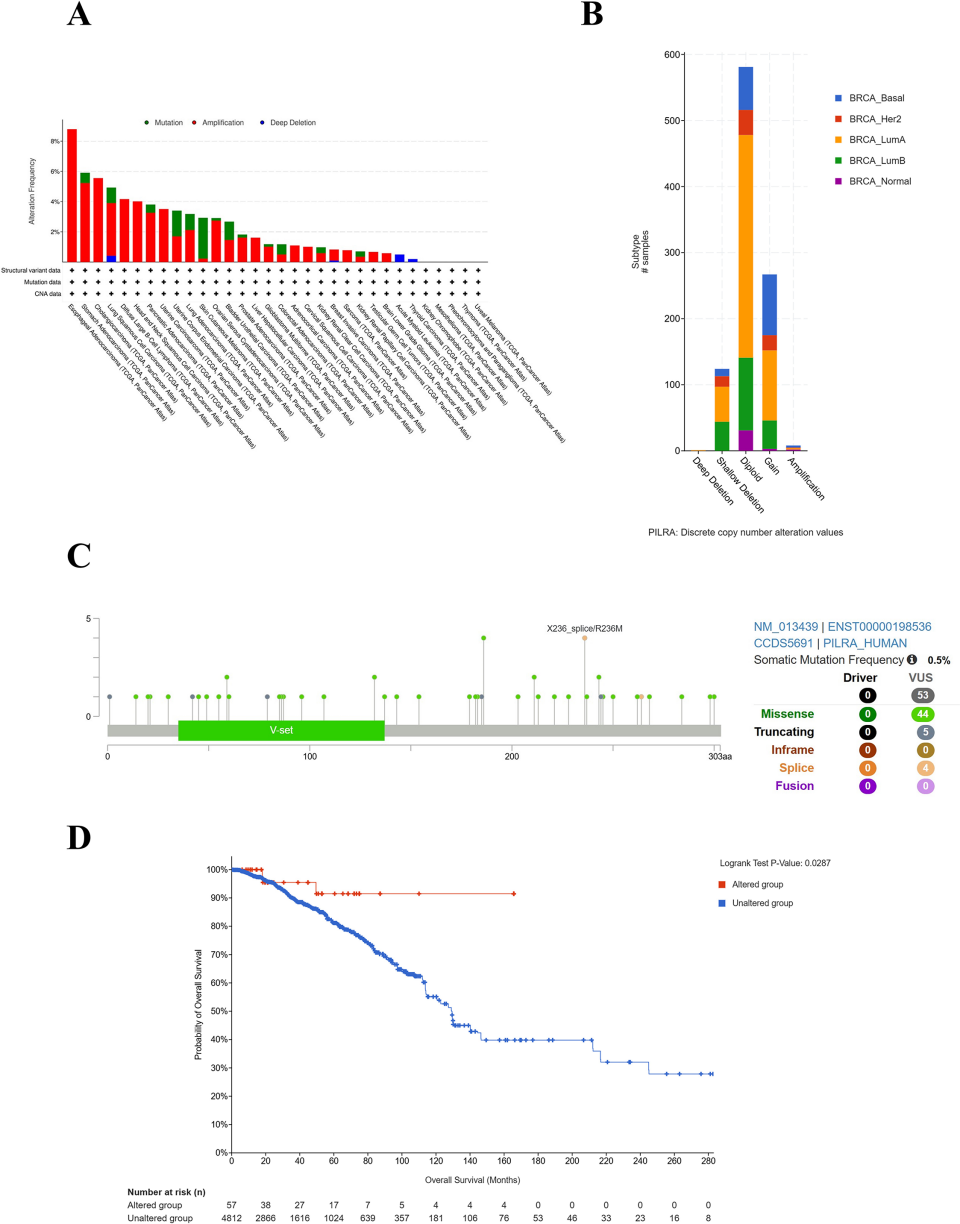
Genetic alterations of PILRA in breast cancer and association with breast cancer patient prognosis. **(A)** Bar charts displaying the genetic alteration frequency of PILRA in different cancer types according to the cBioPortal database. The alterations include mutation, amplification, and deep deletion. **(B)** Bar charts displaying the genetic alteration frequency of PILRA in breast cancer subtypes. **(C)** Mutation sites of PILRA spanning its protein domains, including missense, truncating, in-frame, splice, and SV/fusion mutations. **(D)** Associations between PILRA genetic alterations and prognosis in breast cancer.

The mutation profiles of PILRA in different cancer types were analyzed using the TCGA datasets via cBioPortal; three primary genetic alterations were analyzed: amplification, deep deletion, and mutation. As illustrated in **Figure 2A**, the mutation rate of the PILRA gene was highest in esophageal adenocarcinoma, reaching 8.79%, whereas a lower mutation rate of 0.83% was observed in breast invasive carcinoma (of 1084 cases). Amplification was the most prevalent alteration of PILRA, showing a higher frequency than that of mutation and deep deletion in different cancers. Among the breast cancer subtypes, Luminal A exhibits the highest frequency of PILRA alterations, with diploid events being the predominant alteration type (**Figure 2B**). As depicted in the mutation diagram, 53 mutation sites were identified in PILRA, and R236M was the predominant mutation site (**Figure 2C**). Because genetic alterations can correlate with clinical survival outcomes, we compared survival differences between PILRA-altered and PILRA-unaltered groups (**Figure 2D**). The results indicate that the presence of PILRA alterations was significantly associated with improved prognosis in breast cancer.

### 3.3 PILRA expression is associated with drug resistance in breast cancer

**Figure 3:**
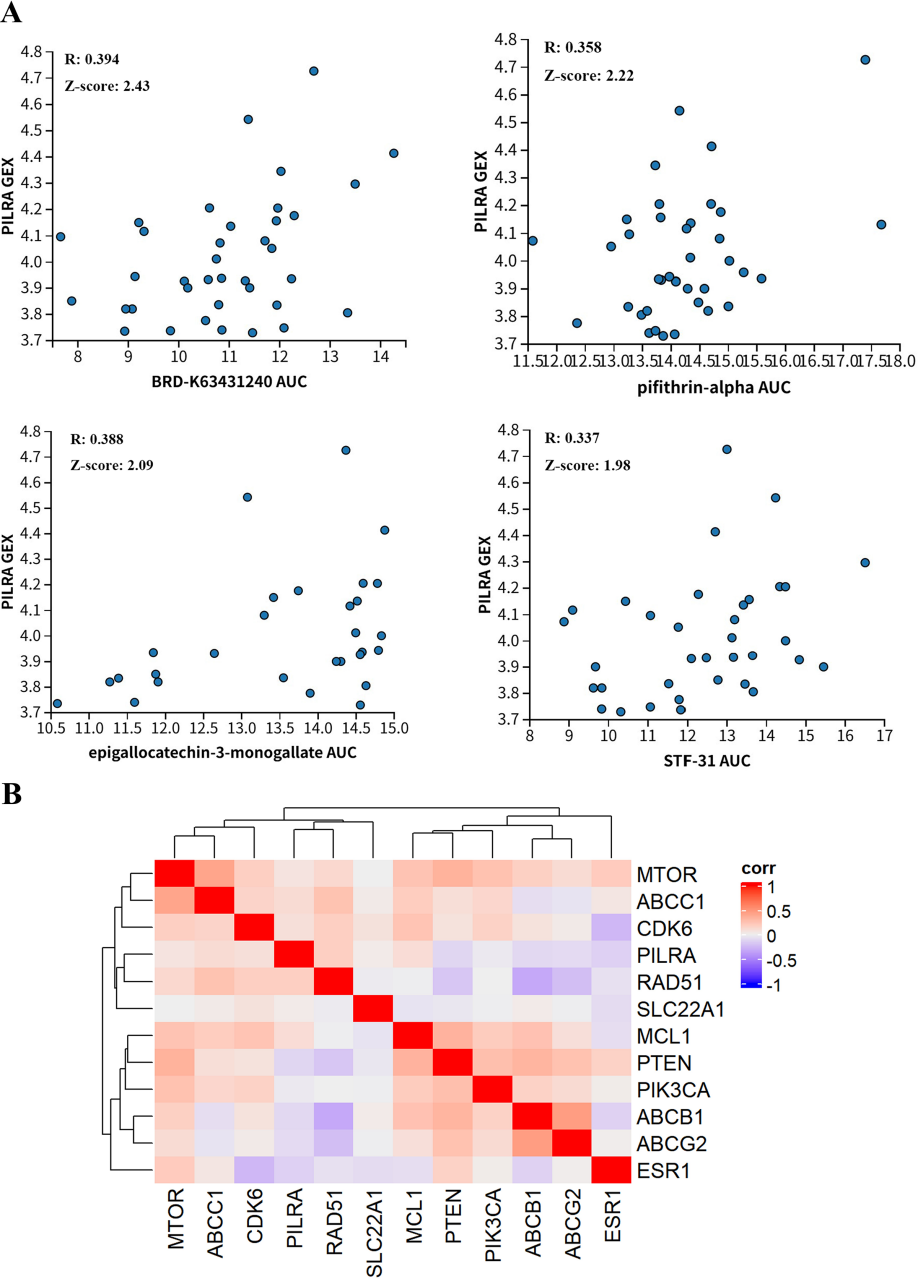
Relationship between PILRA expression and drug resistance in breast cancer. **(A)** Correlation between PILRA expression and drug response (AUC) for four compounds in breast cancer. **(B)** Cross−heatmap illustrating the co−expression patterns of PILRA with drug resistance−related genes.

The association between PILRA expression and drug response across breast cancer cell lines was explored using the Cancer Therapeutics Response Portal (CTRP) dataset (**Figure 3A**). Drug sensitivity was evaluated by the area under the dose–response curve (AUC). Correlation analysis revealed that PILRA expression was positively correlated with the AUC values of several compounds, including BRD-K63431240, pifithrin-α, epigallocatechin-3-monogallate, and STF-31. These compounds mainly target the p53 signaling pathway (pifithrin-α), glucose metabolism via GLUT1 (STF-31), PI3K/AKT and MAPK signaling (EGCG), and cell survival–related pathways (BRD-K63431240), collectively suggesting that PILRA may be associated with drug resistance–related drug response through multiple, pathway-level mechanisms. Meanwhile, we analyzed the relationships between PILRA expression and multiple drug resistance– and drug sensitivity–related genes involved in drug efflux, cell cycle regulation, anti-apoptotic signaling, DNA damage repair, and the PI3K/AKT/mTOR signaling pathway (21–28). PILRA expression was positively associated with the upregulation of drug resistance–related genes and negatively associated with the expression of drug sensitivity–related genes (**Figure 3B**). Together, these results suggest that PILRA may be involved in breast cancer drug resistance.

### 3.4 PILRA methylation correlates negatively with its expression and associates with improved survival in breast cancer

**Figure 4:**
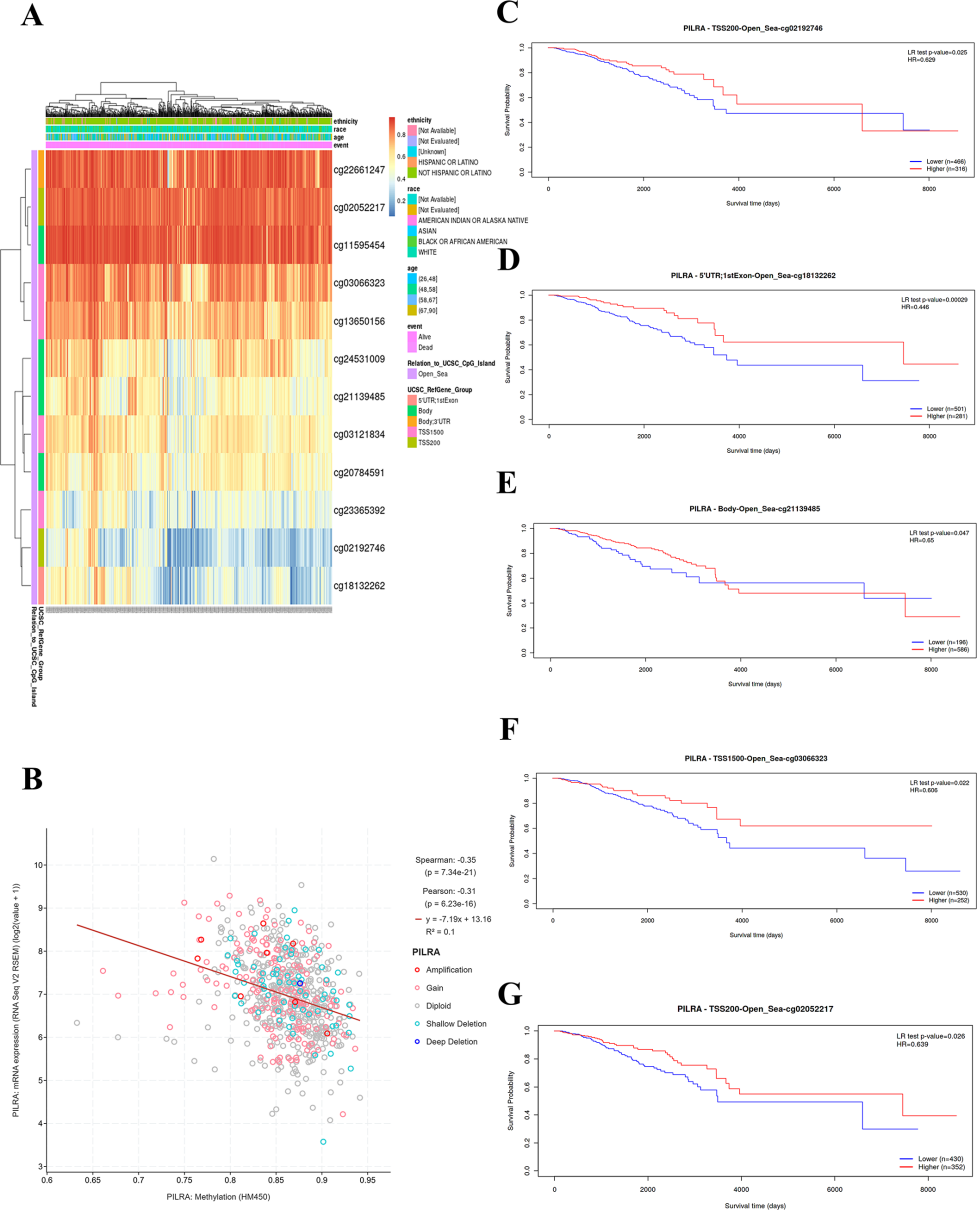
PILRA methylation is inversely correlated with expression and linked to better survival in breast cancer. **(A)** Heat map showing twelve methylated CpG sites in PILRA (Values range from 1 (fully methylated, red) to 0 (fully unmethylated, blue). Methylation data were obtained from the MethSurv database. **(B)** Correlations between the gene expression level and methylation of PILRA in breast cancer samples. **(C–G)** Survival curves based on selected CpG methylation sites.

DNA methylation is a crucial epigenetic mark that modulates gene expression. We identified 12 methylation sites in PILRA in breast cancer (**Figure 4A**), among which CG22661247 and CG02052217 were the most methylated sites. Additionally, we observed a significant negative correlation between PILRA methylation and its expression levels (**Figure 4B**). Analysis of the relationship between PILRA methylation sites and survival in breast cancer patients showed that PILRA methylation significantly improved patient survival (**Figures 4C–G**).

### 3.5 PILRA expression predicts poor survival and moderate diagnostic accuracy in breast cancer

**Figure 5:**
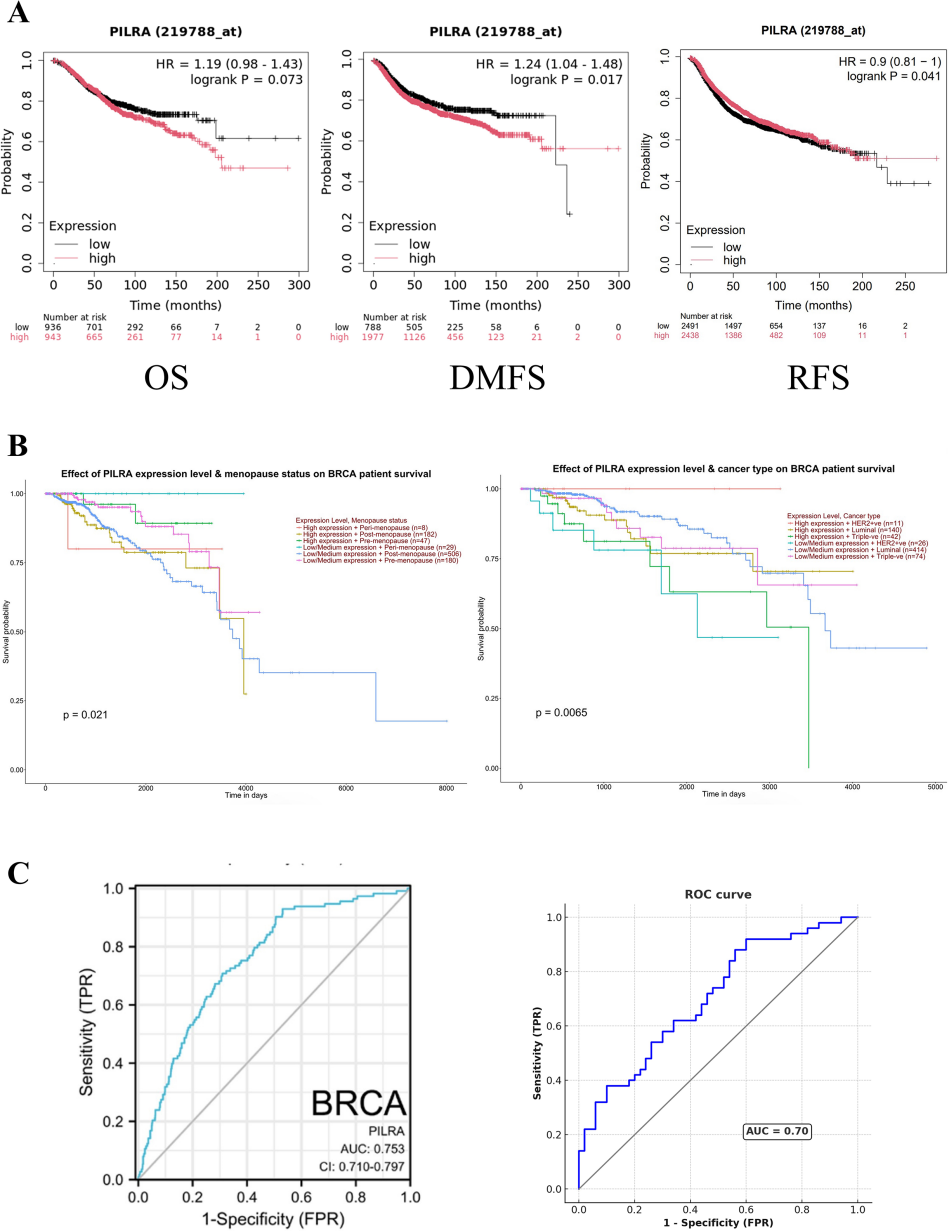
PILRA expression is associated with poor survival and demonstrates good diagnostic accuracy in breast cancer. **(A)** Kaplan–Meier curves for overall survival (OS), relapse-free survival (RFS), and distant metastasis-free survival (DMFS). **(B)** PILRA expression influenced survival outcomes in various cancer types and different menopause statuses. **(C)** ROC curve analysis evaluating the diagnostic potential of PILRA in breast cancer based on TCGA datasets and tissue samples from fifty breast cancer patients.

To evaluate the prognostic value of PILRA gene expression in breast cancer patients, we generated and analyzed Kaplan–Meier curves for OS, RFS, and DMFS (**Figure 5A**). High PILRA expression was significantly associated with poor RFS and DMFS, whereas the impact of PILRA expression on OS was not statistically significant. Elevated PILRA expression was associated with reduced survival in breast cancer patients with different cancer types and menopause status (**Figure 5B**). Next, the diagnostic value of PILRA in breast cancer was evaluated using the TCGA dataset and expression data from fifty paired cancer and adjacent normal tissue samples. As illustrated by the ROC curves (**Figure 5C**), PILRA expression showed moderate diagnostic efficacy in breast cancer (0.9 > AUC > 0.7). Collectively, these findings indicate that PILRA may serve as a potential diagnostic biomarker in breast cancer.

### 3.6 PILRA exhibits heterogeneous expression in breast cancer and immune cells and influences breast cancer cell proliferation

**Figure 6:**
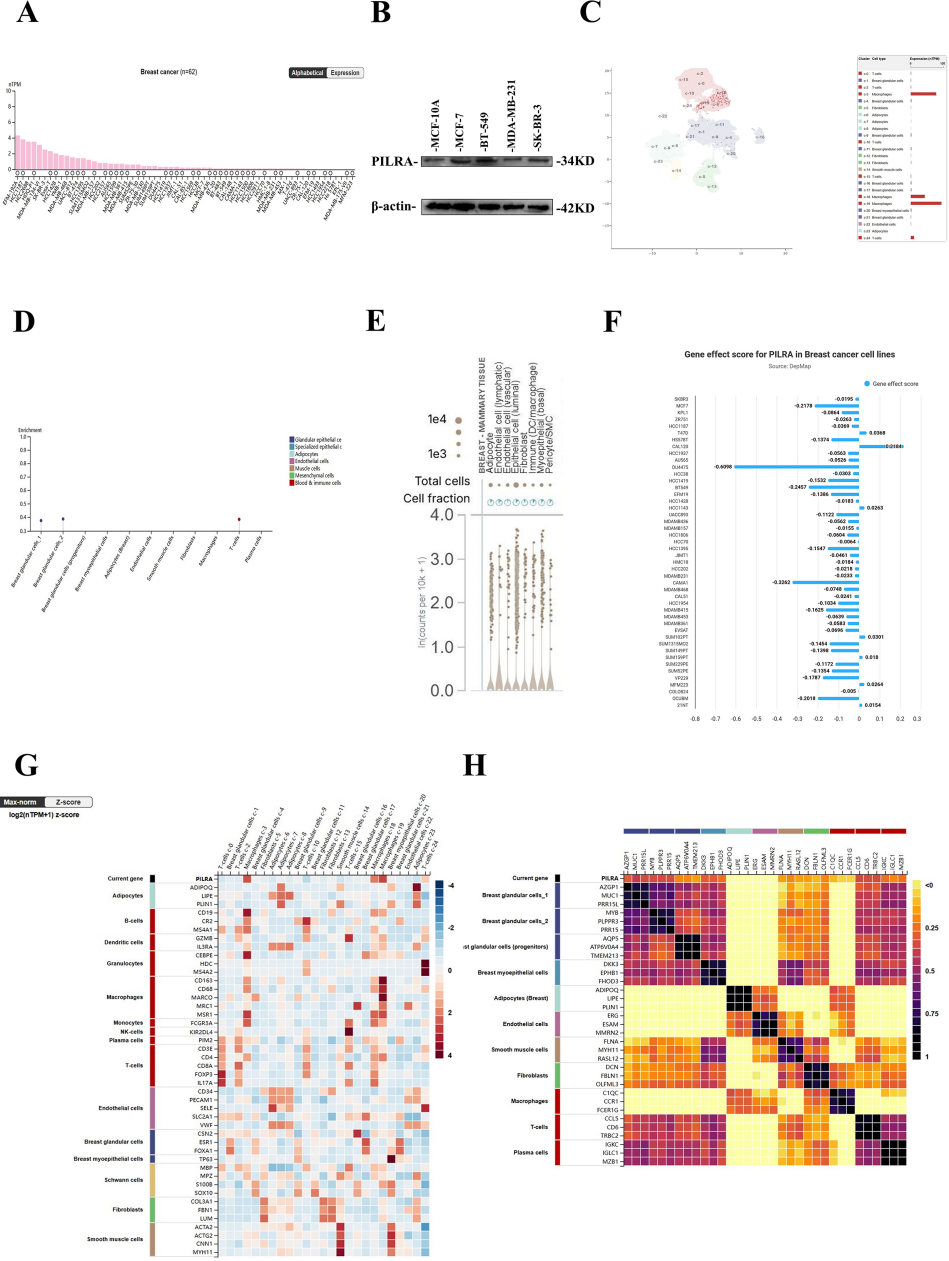
PILRA expression varies across breast cancer cell lines and immune cells, with knockout inhibiting cancer cell growth. **(A)** Expression of PILRA in breast cancer cell lines. **(B)** Western blot analysis of PILRA expression in breast cancer cell lines. **(C)** UMAP visualization of RNA expression in identified single-cell clusters of breast tissue. **(D, E)** Enrichment analysis of PILRA in breast tissue cell types. **(F)** Gene effect score for PILRA in breast cancer cell lines. **(G)** Single-cell marker analysis of PILRA in breast tissue. **(H)** Tissue cell marker analysis of PILRA in breast tissue.

PILRA expression was notably elevated in breast cancer cell lines (**Figure 6A**), particularly in EFM192A and HCC1187 cells, indicating that PILRA expression varies across different breast cancer cell lines, potentially reflecting cell line-specific characteristics or breast cancer subtypes. Meanwhile, Western blot analysis was performed to validate PILRA expression in several breast cancer cell lines(**Figure 6B**). Single-cell analysis revealed that PILRA was highly expressed in macrophages and T cells (**Figure 6C**), suggesting its potential involvement in the immune microenvironment of breast cancer. Tissue cell analysis revealed elevated PILRA expression in luminal epithelial cells, myoepithelial cells, and immune cells (**Figures 6D, E**), suggesting its potential role in maintaining tissue structure, regulating secretion, and providing cellular support in breast tissue. These results suggest that PILRA has multifunctional roles in different cell types and is potentially linked to immune regulation and tissue function. We also analyzed the gene effect scores of PILRA in breast cancer cell lines; negative scores indicate cell growth inhibition and/or cell death following gene knockout. PILRA knockout resulted in varying degrees of growth inhibition in most breast cancer cell lines, suggesting its potential role in maintaining cancer cell proliferation (**Figure 6F**). Marker analysis from both single-cell and tissue cell studies confirmed the high expression of PILRA in immune cells, supporting its role in the breast cancer immune microenvironment (**Figures 6G, H**).

### 3.7 PILRA expression associates with immune cell infiltration and varies among breast cancer subtypes

**Figure 7:**
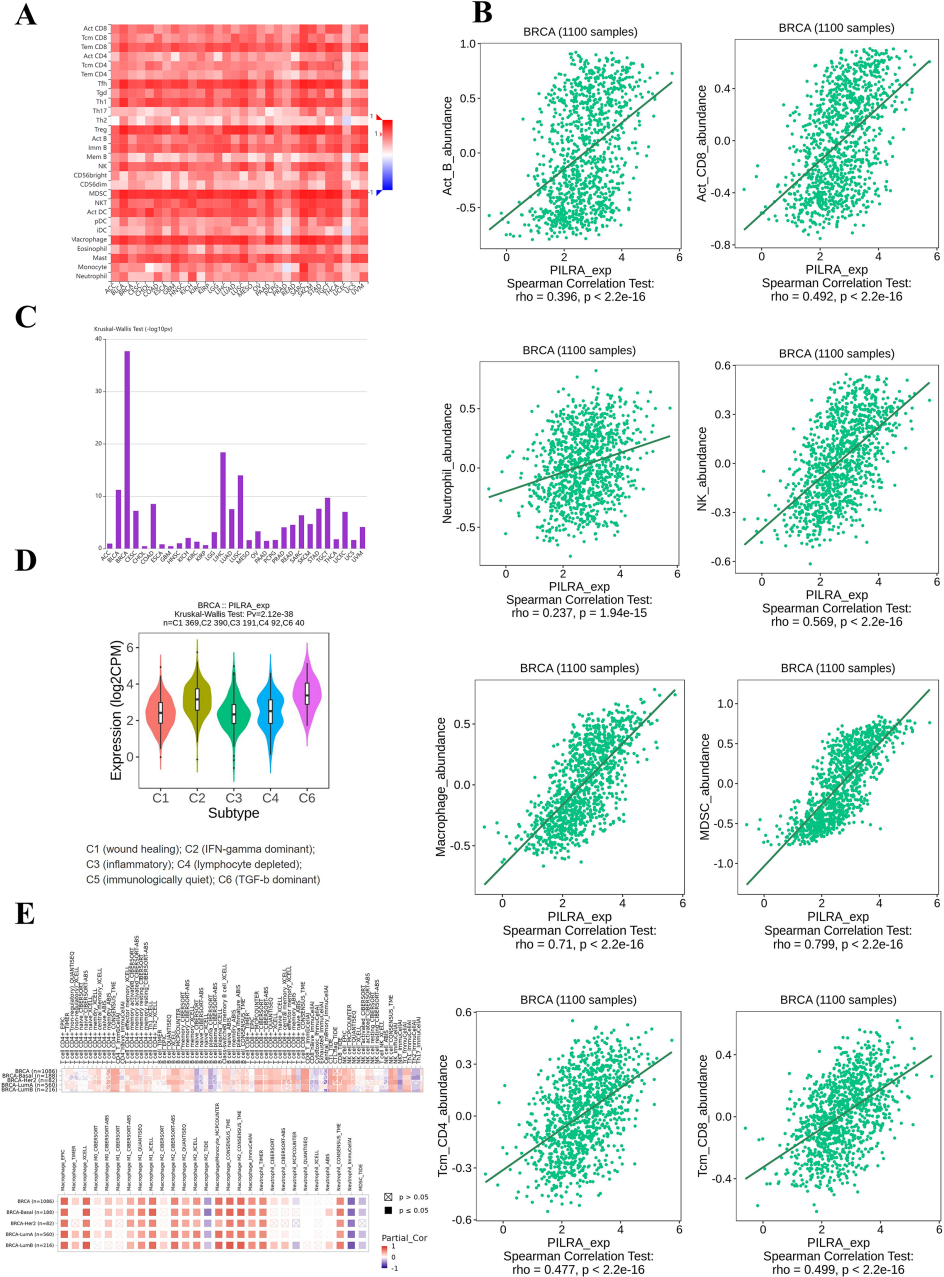
PILRA expression is positively linked to immune cell infiltration and displays subtype-specific patterns in breast cancer. **(A)** The impact of PILRA expression on the abundance of major immune cell subtypes in multiple cancer types. **(B)** Correlation between PILRA expression and the abundance of ACTB, ACT CD8, macrophages, MDSCs, neutrophils, NK cells, TCM CD4, and TCM CD8 cells. **(C)** Associations between PILRA expression and immune subtypes in human cancers **(D)** Distribution of PILRA expression among breast cancer immunological subtypes. **(E)** Correlation between PILRA expression and immune cell abundance in different breast cancer molecular subtypes.

Cancer immunotherapy largely relies on the accumulation and activation of immune effector cells within the tumor microenvironment (TME), as a higher infiltration of immune cells is generally indicative of an immuno-supportive TME, which can enhance antitumor immune responses (29). We utilized the TISIDB platform and TCGA database to analyze the impact of PILRA expression on the abundance of major immune cell subtypes in multiple cancer types (**Figure 7A**). PILRA expression was positively correlated with the abundance of most immune cell subtypes in both breast cancer and other cancer types. Furthermore, separate Spearman’s correlation analyses specifically in breast cancer were performed to assess key immune cell types (**Figure 7B**), including CD8+ T cells, macrophages, and natural killer (NK) cells. The results revealed a significant positive correlation between PILRA expression and the abundance of these major immune cell types in breast cancer. The results of the Kruskal-Wallis test indicated that PILRA expression differed significantly between breast cancer and other cancer types, suggesting that PILRA plays a specific role in breast cancer (**Figure 7C**). We also investigated the distribution of PILRA expression across breast cancer subtypes (**Figures 7D, E**). Taken together, the results suggest that PILRA exerts distinct immune functions, particularly in breast cancer and its various immunological subtypes.

### 3.8 PILRA associates with multiple genes, proteins, and disease-related pathways

**Figure 8:**
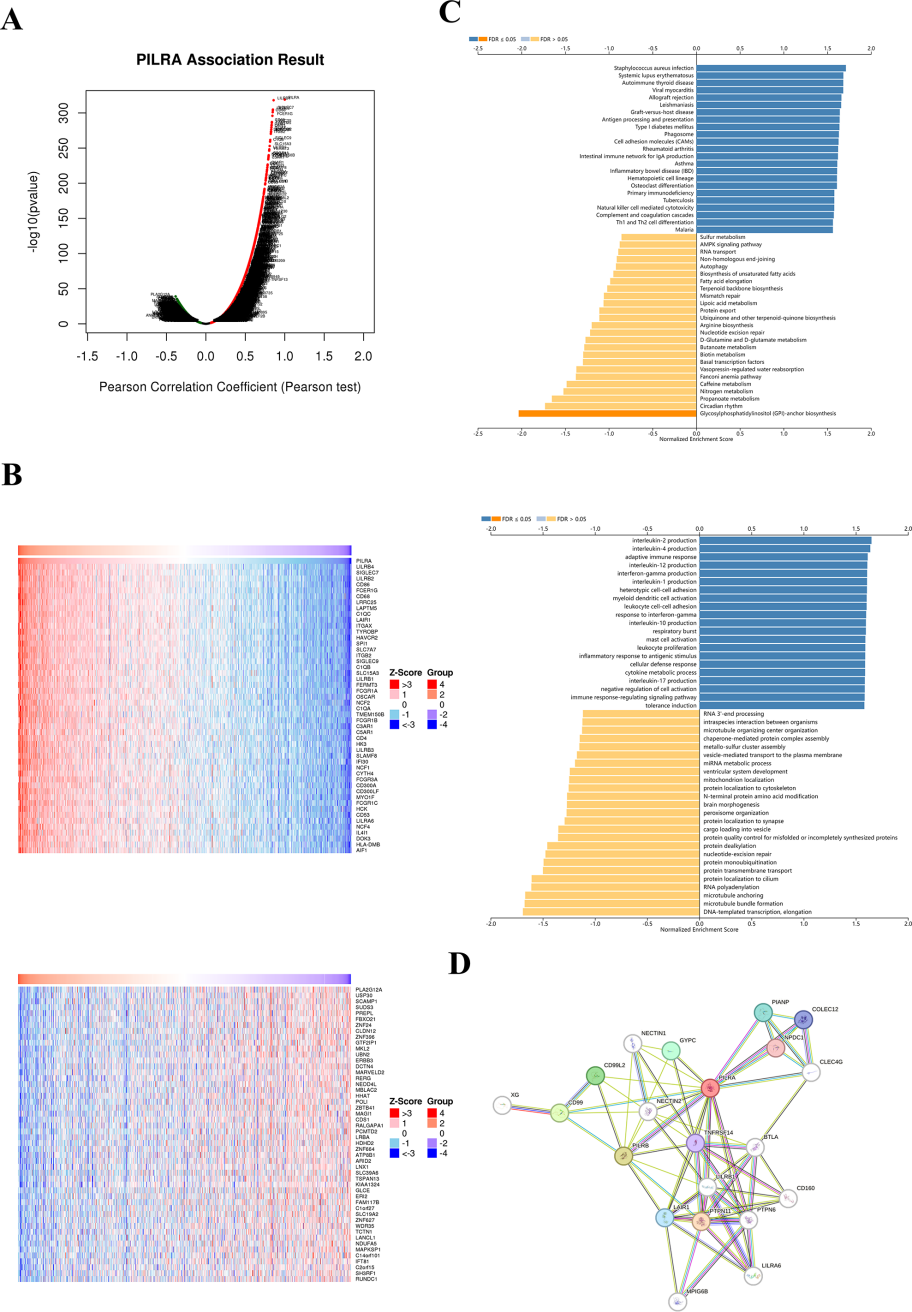
PILRA correlates with genes and proteins and participates in disease-related pathways. **(A)** Gene correlation analysis based on the TCGA-BRCA database. **(B)** Gene heatmaps were generated for the top 50 positively (up) and negatively (down) correlated genes. **(C)** Results of KEGG analysis (up) and GO analysis (down). **(D)** Network of experimentally validated PILRA-interacting partners visualized using the STRING web tool.

We performed gene correlation analysis using the Linkedomics tool in the TCGA-BRCA database, which includes 20,155 genes(**Supplementary Table 1**). The correlation coefficients were obtained using Pearson’s correlation analysis (**Figure 8A**). Gene heatmaps were generated for the top 50 positively and negatively correlated genes (**Figure 8B**). PILRA exhibited the highest positive correlation with genes such as LILRB4 and SIGLEC7, and the highest negative correlation with genes such as PLA2G12A and USP30. The results of KEGG and GO analyses (**Figure 8C**) showed that PILRA was strongly associated with diseases including Staphylococcus aureus infection and systemic lupus erythematosus. PILRA was related to processes involving interleukin-2 and interleukin-4 production. A PPI network of PILRA was constructed (**Figure 8D**), and different colored lines represent distinct types of interactions. Green lines indicate gene neighborhood, red lines represent gene fusions, and blue lines signify gene co-occurrence. The results showed interactions between PILRA and proteins PTPN11, PILRB, and CD99.

## 4 Discussion

Breast cancer poses a significant threat to the physical and mental health of women worldwide. Its incidence is increasing at an annual rate of approximately 3%, with a noticeable trend towards younger patient populations (30). In China, breast cancer is not only one of the most prevalent malignancies, but also a leading cause of cancer-related death among women; its increasing incidence rate poses a serious challenge for prevention and treatment (31). In this study, we identified PILRA as significantly dysregulated in 16 cancer types, including breast cancer, through integrated bioinformatics and experimental validation. Survival and mutation analyses showed that PILRA is associated with poor prognosis in breast cancer patients. Furthermore, PILRA affects immune cell infiltration in breast cancer. KEGG and GO enrichment analyses, together with gene co-expression analysis, shed light on the potential pathways and biological functions related to PILRA.

Assessing gene expression patterns offers valuable insights into the molecular landscape of cancer and enables the identification of candidate biomarkers. For instance, overexpression of the actin-binding protein ANLN is essential for cell cycle progression (32). Cyclin E2 (CCNE2), which is known for its proliferative properties, is associated with genome doubling in breast cancer, and its overexpression induces aneuploidy and genomic instability (33). Similarly, upregulation of kinesin family member 4A (KIF4A) disrupts chromosomal integrity, midzone formation, and cytokinesis regulation (34). In this study, we demonstrated that PILRA, an immune inhibitory receptor, is significantly upregulated in breast cancer tissues compared with normal breast tissues. Analysis of TCGA data, validated by IHC and RT-qPCR, consistently demonstrated higher PILRA expression levels in breast cancer samples, and the difference was statistically significant. These findings suggest the potential of PILRA as a biomarker for breast cancer and provide a basis for further exploration of its biological functions.

Specific gene mutations have prognostic relevance in cancer (35). For example, TP53 mutations are associated with poor overall survival in breast cancer (36), and genetic alterations such as 11q13 gain correlate with unfavorable outcomes in oral squamous cell carcinoma (37). A pan-cancer study also supports that particular driver mutations can carry prognostic value (38). Motivated by these observations, in this study, we found that abnormal mutations in PILRA affected patient survival. Mutation and survival analyses revealed that PILRA exhibits a high alteration frequency in pan-cancer, particularly in breast cancer. Among breast cancer subtypes, Luminal A shows the highest frequency of PILRA alterations. Moreover, PILRA mutations are associated with improved survival outcomes, suggesting a subtype-specific role of PILRA in breast cancer progression and identifying it as a potential prognostic marker. The correlation between PILRA expression and chemoresistance suggests that PILRA plays a role in breast cancer treatment response, making it a potential biomarker for patient prognosis. A significant negative correlation between PILRA methylation and mRNA expression suggests that methylation regulates PILRA expression. Additionally, higher methylation levels were associated with improved survival in breast cancer, indicating the potential prognostic value of PILRA methylation. To further explore this, we conducted extensive survival analyses including OS, RFS, DMFS, and subtype-specific survival analysis. ROC curves confirmed that PILRA may serve as a reliable prognostic biomarker for breast cancer.

This study provides a novel perspective on the role of PILRA in breast cancer immunotherapy. Previous research focused on the immunoregulatory function of PILRA, such as its role as a gB-associated co-receptor in monocyte HSV-1 infection (39). The PILRA G78R variant has been linked to a significant reduction in HSV-1 infection levels in macrophages (40). However, the immunomodulatory role of PILRA in breast cancer remains unexplored. In this study, we found that PILRA expression was positively correlated with tumor immune infiltration in breast cancer, particularly across different subtypes, as well as in other cancer types. Furthermore, PILRA expression was positively correlated with the abundance of various immune cell subtypes, including CD8+ T cells, macrophages, and natural killer cells. The high expression levels of PILRA in breast cancer cell lines and immune cells suggests its potential role in the immune microenvironment of breast cancer. Single-cell and tissue cell analyses further support the multifunctional role of PILRA in immune regulation and tissue function, indicating its potential as a biomarker for immunotherapy. These findings strongly suggest that PILRA is involved in modulating cancer-associated immune processes and could play a significant role in immunotherapy.

Gene co-expression and KEGG/GO analyses revealed that PILRA is highly implicated in the pathogenesis of other diseases and may influence several cancer-related pathways, including the regulation of interleukin-2 and interleukin-4 production. These findings point to potential avenues for future research.

In conclusion, our integrated analysis demonstrates that PILRA is significantly overexpressed in breast cancer and is associated with poor clinical outcomes. Moreover, its correlation with immune infiltration and potential involvement in multiple immune-related pathways highlights its value as a diagnostic, prognostic, and therapeutic target in breast cancer.

## Data Availability

The original contributions presented in the study are included in the article/**Supplementary Material**. Further inquiries can be directed to the corresponding authors.
